# Supplementary material for: Bacterial vampirism mediated through taxis to serum
Source: eLife. 2024 May 31;12:RP93178. doi: 10.7554/eLife.93178 (PMC11142651; doi:10.7554/eLife.93178)
Supplement: Supplementary file 1. [file elife-93178-supp1.docx]

| Table of crystallographic statistics | |  | | |  | |  |  |
| --- | --- | --- | --- | --- | --- | --- | --- | --- |
| Protein, PDB code | *Se*Tsr LBD (pH 7.5-9.7)  PDB: 8FYV | *Se*Tsr LBD (pH 7-7.5)  PDB: 8VL8 | | |  |  |  |  |
| Space Group | C 1 2 1 | C 1 2 1 | | |  |  |  |  |
| Cell dimensions and angle  (a, b, c, beta) (Å, º) | 125.8, 75.2, 129.6, 116.5 | 126.0, 74.8, 128.9, 116.1 | | |  |  |  |  |
| Resolution (Å) ^a^ | 58.0-2.2 (2.26-2.20) | 62.4-2.12 (2.18-2.12) ^b^ | | |  |  |  |  |
| Completeness (%) ^a^ | 100.0 (100.0) | 98.3 (88.5) | | |  |  |  |  |
| Total reflections | 1,039,561 | 367,890 | | |  |  |  |  |
| Unique reflections | 55,162 | 60,193 | | |  |  |  |  |
| Average *I/σ* ^a^ | 7.7 (3.6) | 5.4 (2.0) | | |  |  |  |  |
| CC_1/2_ ^a^ | 0.994 (0.838) | 0.977 (0.568) | | |  |  |  |  |
| R_work_ (%) | 24.1 | 22.7 ^b^ | | |  |  |  |  |
| R_free_ (%) | 25.8 | 27.4 ^b^ | | |  |  |  |  |
| Ramachandran favored  , allowed, outliers (%) | 99.1, 0.9, 0.0 | 99.6, 0.4, 0.0 | | |  |  |  |  |
| Protein non-hydrogen atoms | 5604 | 5617 | | |  |  |  |  |
| Solvent atoms | 283 | 224 | | |  |  |  |  |
| Protein chains, residues | 5, 705 | 5, 705 | | |  |  |  |  |
| Average B-factor of  protein atoms (Å^2^) | 49 | 35 | | |  |  |  |  |
| Average B-factor  of solvent atoms (Å^2^) | 43 | 37 | | |  |  |  |  |
| rms bond lengths (Å) | 0.002 | 0.013 | | |  |  |  |  |
| rms bond angles (°) | 0.42 | 1.22 | | |  |  |  |  |
| ^a^ Values in parentheses indicate statistics for the highest resolution shell.  ^b^ Refinement was restricted to 2.5 Ǻ resolution. |  |  | | |  |  |  |  |
|  | |  | | |  |  | | |
|  | | |  |  |  |  | |  |
